# Long noncoding RNA MIAT inhibits the progression of diabetic nephropathy and the activation of NF-κB pathway in high glucose-treated renal tubular epithelial cells by the miR-182-5p/GPRC5A axis

**DOI:** 10.1515/med-2021-0328

**Published:** 2021-09-06

**Authors:** Qianlan Dong, Qiong Wang, Xiaohui Yan, Xiaoming Wang, Zhenjiang Li, Linping Zhang

**Affiliations:** Kidney Disease and Dialysis Center, Shaanxi Provincial People’s Hospital, Beilin District, Xi’an, Shaanxi, 710068, China

**Keywords:** MIAT, DN, miR-182-5p, GPRC5A, NF-κB pathway

## Abstract

**Background:**

Diabetic nephropathy (DN) is a common diabetic complication. Long noncoding RNAs (lncRNAs) have been identified as essential regulators in DN progression. This study is devoted to the research of lncRNA-myocardial infarction-associated transcript (MIAT) in DN.

**Methods:**

DN cell model was established by high glucose (HG) treatment for human renal tubular epithelial cells (HK-2). Cell viability and colonizing capacity were analyzed by Cell Counting Kit-8 (CCK-8) and colony formation assay. Apoptosis was assessed via caspase-3 detection and flow cytometry. Enzyme-linked immunosorbent assay (ELISA) was used for evaluating inflammation. The protein determination was completed using western blot. MIAT, microRNA-182-5p (miR-182-5p), and G protein-coupled receptor class C group 5 member A (GPRC5A) levels were all examined via reverse transcription-quantitative polymerase chain reaction (RT-qPCR). Intergenic binding was verified using dual-luciferase reporter, RNA immunoprecipitation (RIP), and RNA pull-down assays.

**Results:**

HG induced the inhibition of cell growth, but accelerated apoptosis and inflammation as well as the activation of nuclear factor kappa B (NF-κB) pathway. MIAT reestablishment prevented the HG-induced cell damages and NF-κB signal activation. Mechanistically, MIAT was proved as a miR-182-5p sponge and regulated the expression of GPRC5A that was a miR-182-5p target. The rescued experiments demonstrated that MIAT downregulation or miR-182-5p upregulation aggravated the HG-induced cell damages and activated the NF-κB pathway via the respective regulation of miR-182-5p or GPRC5A.

**Conclusion:**

Taken together, MIAT functioned as an inhibitory factor in the pathogenesis to impede the development of DN and inactivate the NF-κB pathway via regulating the miR-182-5p/GPRC5A axis.

## 1 Introduction

Diabetic nephropathy (DN), the most familiar microvascular complication in diabetic type 1 and type 2 patients with high incidence rate, is considered as the leading risk factor of chronic kidney failure and end-stage renal diseases [1,2]. The typical pathological feature for DN is the damage of capillaries in the glomeruli, and the inflammation formation has also been involved in the pathological progression of DN [3]. The molecular pathways have become the new insights in the therapeutic paradigms of DN [4], especially long noncoding RNAs (lncRNAs) and microRNAs (miRNAs) [5,6].

lncRNAs (>200 nucleotides) are one common family of regulatory ncRNAs in diverse biological processes of diseases [7], such as osteoarthritis [8], cardiovascular disease [9], and liver disease [10]. lncRNAs have also been regarded as important regulators in the regulation of DN [11,12]. PVT1 silence reduced cell proliferation, migration, and fibrosis in DN cell model [13]; NEAT1 acted as a pro-inflammatory molecule in DN [14]. Myocardial infarction-associated transcript (MIAT) downregulation has been correlated to the renal tubular epithelial injury in DN [12], and MIAT could enhance cell viability in high glucose (HG)-treated renal tubular cells by increasing the expression of Nrf2 [15]. A recent study reported that the antagonistic effect of MIAT on DN progression was related to the sponge function on miR-147a and the expression regulation on E2F3 [16]. It is unknown whether the regulation of MIAT in DN progression is related to other molecular mechanism.

miRNAs are short ncRNAs (∼22 nucleotides) with pivotal effects on the epigenetic modifications of DN [17]. Meta-analysis has identified that miR-126 and miR-770 could be used as noninvasive biomarkers for DN [18], and miR-184 could govern renal fibrosis in DN [19]. lncRNAs can exert the “sponge-like” effects on various miRNAs to limit the miRNA-mediated actions in human diseases [20]. Ming et al. reported that microRNA-182-5p (miR-182-5p) contributed to the pathogenetic development to serve as a risk factor in DN [21]. It remains unclear whether MIAT has a sponge effect on miR-182-5p.

G protein-coupled receptor class C group 5 member A (GPRC5A) is a sub-member of G protein-coupled receptors (GPCRs) superfamily. GPRC5A regulated the progression of human diseases and it was associated with many signaling pathways, such as nuclear factor kappa B (NF-κB) pathway [22]. GPRC5A overexpression could accentuate the HG-induced renal proximal tubule cell injury and it functioned as a target of miR-218 [23]. miRNAs have been implicated in the epigenetic modulation of DN by binding to the 3′-untranslated regions (3′-UTRs) of mRNAs [24]. The target relation between GPRC5A and miR-182-5p will be explored in this study.

Herein the effects of MIAT on other cellular processes of DN were further researched. More importantly, we have disclosed the regulatory network among MIAT, miR-182-5p, and GPRC5A.

## 2 Materials and methods

### 2.1 Cell culture and HG induction

Human renal tubular epithelial cell line (HK-2) was bought from American Type Culture Collection (ATCC, Manassas, VA, USA) and sustained in Dulbeccoʼs modified Eagleʼs medium (DMEM)/F12 (Gibco, Carlsbad, CA, USA) with the supplement of 10% of fetal bovine serum (FBS; Gibco) and 1% of penicillin/streptomycin solution (Gibco). Cell culture was performed in a 37°C humid incubator containing 5% of CO_2_. DN cell model was established by treating HK-2 cells with HG (25 mM of glucose) for 48 h, using the normal glucose (NG; 0 mM of glucose) and low glucose (LG; 5 mM of glucose) as the control treatment groups.

### 2.2 Cell Counting Kit-8 (CCK-8) assay

CCK-8 (Dojindo, Kumamoto, Japan) was applied for assessing the cell viability. In the 96-well plates, the treated HK-2 cells were added with 10 μL/well CCK-8 solution. Following incubation for 3 h, the absorbance was assayed at 450 nm via a microplate reader (Bio-Rad, Hercules, CA, USA).

### 2.3 Colony formation assay

Cell inoculation was conducted at 48 h post-transfection with 500 cells/well in 6-well plates. Cells were cultivated for 2 weeks under the standard culture condition. Subsequently, the colonies were fastened with methanol (Sigma-Aldrich, St. Louis, MO, USA) for 10 min and stained with crystal violet (Sigma-Aldrich) for 15 min. These colonies were photographed by a digital camera and the colonizing number was counted.

### 2.4 Caspase-3 activity detection

Total proteins were obtained via RIPA Lysis buffer (Thermo Fisher Scientific, Waltham, MA, USA) and caspase-3 activity was measured via the EnzChek^®^ Caspase-3 Assay Kit (Invitrogen, Carlsbad, CA, USA) using Z-DEVD-AMC substrate, in compliance with the supplied manual for users.

### 2.5 Flow cytometry

At 72 h post-transfection, apoptosis detection was carried out through the double staining of Annexin V-FITC and propidium iodide (PI) using Apoptosis Detection Kit (Dojindo) as per the operating procedures of the producer. Through the analysis on the flow cytometer (FacsCanto II; BD Biosciences, San Diego, CA, USA), the apoptotic cells were distinguished as the stained cells with Annexin V(+)/PI(−) and Annexin V(+)/PI(+). The common formula: apoptotic cells/total cells × 100% was used to calculate the apoptotic rate.

### 2.6 Enzyme-linked immunosorbent assay (ELISA)

Interleukin-6 (IL-6), tumor necrosis factor-alpha (TNF-α), and Interleukin-1beta (IL-1β) levels were measured via Human ELISA Kits for IL-6, TNF-α, and IL-1β (Invitrogen) according to the instruction books of the manufacturer.

### 2.7 Western blot

Total proteins were produced by lysing cells in RIPA buffer (Thermo Fisher Scientific), followed by detecting the concentration using BCA Protein Assay Kit (Invitrogen). In this study, an equal amount of proteins (30 µg) were applied to perform the western blot analysis, as reported earlier [25]. The antibodies contained primary antibodies against inflammatory NOD-like receptor with a pyrin domain 3 (NLRP3; Cell Signaling Technology (CST), Boston, MA, USA, #13158, 1:1,000), IL-1β (CST, #12703, 1:1,000), NF-κB-associated p65 (CST, #8242, 1:1,000), phospho-p65 (CST, #3033, 1:1,000), GPRC5A (Abcam, Cambridge, UK, ab155557, 1:1,000), and glyceraldehyde-phosphate dehydrogenase (GAPDH; CST, #8884, 1:1,000) and secondary antibody anti-rabbit IgG, HRP-linked Antibody (CST, #7074, 1:2,000). After protein presentation by SignalFire™ ECL Reagent (CST) in the dark, Image Lab software version 4.1 (Bio-Rad) was used for level analysis of the objective proteins with GAPDH as a normalized control.

### 2.8 Cell transfection

The overexpression vectors pEXP-RB-Mam-MIAT (MIAT) and pcDNA-GPRC5A (GPRC5A) were constructed using the basic vectors pEXP-RB-Mam (RIBOBIO, Guangzhou, China) and pcDNA (Invitrogen). Small interfering RNA targeting MIAT (si-MIAT-1: 5′-GGAAUUUCCAGGUUCUUCAGG-3′, si-MIAT-2: 5′-CAUCUUUGCAGAUAAGUAUUU-3′, and si-MIAT-3: 5′-GUGAUGUAGCUCAUUCUCUUU-3′), miR-182-5p mimic and inhibitor (miR-182-5p: 5′-AGUGUGAGUUCUACCAUUGCCAAA-3′ and anti-miR-182-5p: 5′-UCACACUCAAGAUG-GUAACGGUUU-3′), and the control oligonucleotides (si-NC: 5′-UUCUCCGAACGUGUCACGU-3′, miR-NC: 5′-UUCUCCGAACGUGUCACGU-3′, and anti-NC: 5′-CAGUACUUUUGUGUAGUACAA-3′) were directly acquired from RIBOBO. Then, different transfections were implemented via Lipofectamine^™^ 3000 Reagent (Invitrogen) according to the users’ guidelines. The concentration of plasmid or RNA in transfection is shown as below: 2 μg of plasmid (MIAT, vector, GPRC5A, and pcDNA), 40 nM of siRNA (si-MIAT#1/#2/#3 and si-NC), 40 nM of mimic (miR-182-5p and miR-NC), and 20 nM of inhibitor (anti-miR-182-5p and anti-NC).

### 2.9 Reverse transcription-quantitative polymerase chain reaction (RT-qPCR)

Transfected cells were harvested for RNA isolation using Trizol reagent (Invitrogen). Referring to the manufacturer’s directions, the complementary DNA (cDNA) was obtained using RevertAid RT Reverse Transcription Kit (Thermo Fisher Scientific) and RT-qPCR reaction was carried out via SYBR-green PCR Master Mix (Applied Biosystems, Foster City, CA, USA) on the ABI7500 Fast Real-time PCR System (Applied Biosystems). The comparative cycle threshold (2^−∆∆Ct^) method was used to calculate the relative expression levels using GAPDH (for MIAT and GPRC5A) and U6 (for miR-182-5p) as the internal references. Primer sequences were shown as below: MIAT (sense: 5′-GGACGTTCACAACCACACTG-3′, antisense: 5′-TCCCACTTTGGCATTCTAGG-3′); miR-182-5p (sense: 5′-GCCGAGTTTGGCAATGGTAG-3′, antisense: 5′-CGCAGGGTCCGAGGTAT-3′); GPRC5A (sense: 5′-CTCACTCTCCCGATCCTCGT-3′, antisense: 5′-CAGTCCGATGATGAAGGCGAA-3′); GAPDH (sense: 5′-CACCCACTCCTCCACCTTTG-3′, antisense: 5′-CCACCACCCTGTTGCTGTAG-3′); and U6 (sense: 5′-GCTTCGGCAGCACATA-3′, antisense: 5′-ATGGAACGCTTCACGA-3′).

### 2.10 Dual-luciferase reporter assay

The miR-182-5p binding sites in the MIAT or 3′-UTR of GPRC5A sequence (wild-type, WT) were mutated and the mutated MIAT or 3′-UTR of GPRC5A sequence was defined as the mutant-type (MUT). These sequences were cloned into the downstream of luciferase reporter gene in the pGL3-control vector (Promega, Madison, WI, USA) to construct the novel luciferase vectors (WT-MIAT, MUT-MIAT, WT-GPRC5A 3′-UTR, and MUT-GPRC5A 3′-UTR). Then, cell transfection of each of the above vectors with miR-182-5p or miR-NC was performed for 48 h. The dual-luciferase reporter assay system (Promega) was adopted for the subsequent luciferase activity determination following the user protocols. The relative luciferase activity was exhibited using the normalized firefly activity by renilla luciferase activity.

### 2.11 RNA immunoprecipitation (RIP) assay

RIP assay was conducted by Imprint^®^ RIP Kit (Sigma-Aldrich). In brief, 1 × 10^6^ HG-treated HK-2 cells were lysed in RIP lysis buffer and Protein A magnetic beads were pre-coated with antibodies targeting Argonaute-2 (anti-Ago2) or immunoglobulin G (anti-IgG). Cell lysates were then incubated with the antibody-coated magnetic beads at 4°C overnight. The proteins were removed by proteinase K and total RNA was purified by Trizol, followed by the expression analysis of MIAT and miR-182-5p via RT-qPCR as per the above description.

### 2.12 Biotinylated RNA pull-down assay

Biotinylated miR-182-5p mimic (Bio-miR-182-5p; RIBOBIO) and negative Bio-miR-NC control were respectively transfected into cells for 48 h. Whereafter, cell lysates were incubated with streptavidin magnetic beads (Thermo Fisher Scientific) at 4°C overnight and the combined RNA was isolated from the magnetic beads. Then, RT-qPCR was performed to analyze the enrichment of MIAT.

### 2.13 Statistical analysis

Three independent experiments were conducted with three repetitions each time, and the mean value ± standard deviation (SD) was used for data manifestation. Statistical analysis was completed by SPSS 24.0 (IBM Corp., Armonk, NY, USA) and figures were obtained via Graphpad Prism 7 (GraphPad Inc., La Jolla, CA, USA). Student’s *t*-test for two groups and one-way analysis of variance (ANOVA) followed by Tukey’s test for multiple groups were used for the analysis of difference. Statistically, *P* < 0.05 represented a significant difference.

## 3 Results

### 3.1 HG blocked HK-2 cell growth while inducing apoptosis promotion, inflammatory response, and NF-κB activation

**Figure 1:**
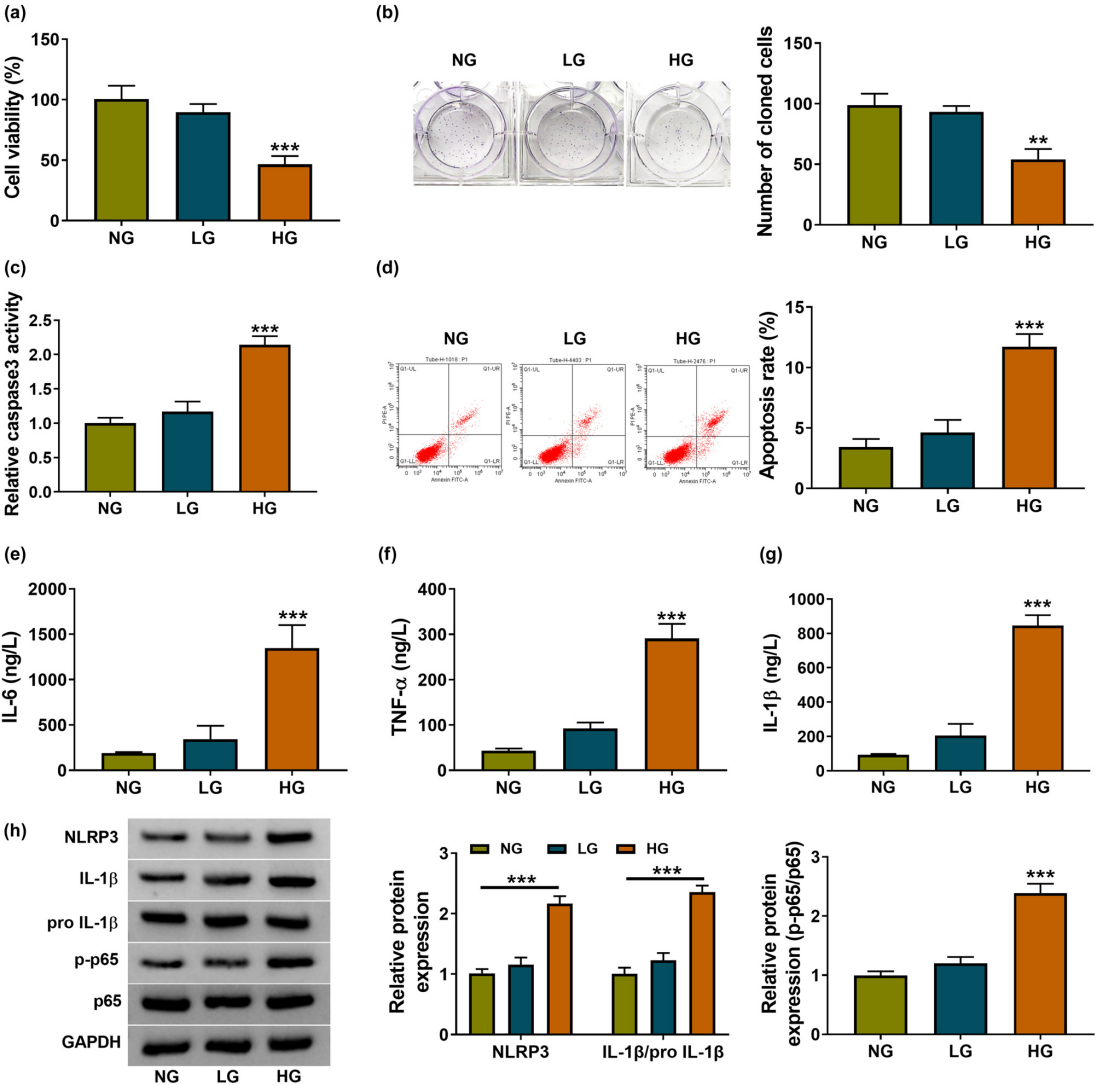
HG blocked HK-2 cell growth while induced apoptosis promotion, inflammatory response, and NF-κB activation. HK-2 cells were treated with NG, LG, or HG for 48 h. (a and b) Cell viability (a) and colonizing capacity (b) were severally measured by CCK-8 and colony formation assay. (c and d) Cell apoptosis was evaluated using caspase-3 activity (c) and apoptosis rate (d). (e–g) ELISA was used to determine the levels of IL-6, TNF-α, and IL-1β. (h) NLRP3, IL-1β/pro-IL-1β, and p-p65/p65 protein levels were assayed via western blot. **P < 0.01, ***P < 0.001.

To explore the effects of HG on HK-2 cells, we analyzed various biological processes through a series of experiments. In comparison to NG and LG groups, cell viability by CCK-8 (Figure 1a) and colonizing ability by colony formation assay (Figure 1b) were reduced after HG treatment with significant changes. Differently, the increased caspase-3 activity (Figure 1c) and apoptosis rate (Figure 1d) in HG-treated HK-2 cells indicated that cell apoptosis was notably facilitated by HG. Then, the inflammatory cytokines were determined by ELISA. The results exhibited that IL-6, TNF-α, and IL-1β levels in HG-treated HK-2 cells were evidently higher than those in NG- or LG-treated cells, suggesting that HG treatment resulted in the inflammatory response (Figure 1e–g). Western blot indicated that HG evoked the upregulation of NLRP3 and IL-1β/pro-IL-1β (the characteristic proteins of inflammation) as well as NF-κB p-p65/p65 (Figure 1h). HG treatment could lead to cellular apoptosis and inflammation in HK-2 cells to simulate the DN environment *in vitro*, and NF-κB pathway was activated by HG.

### 3.2 MIAT reestablishment antagonized the HG-induced cell damages and HG-activated NF-κB pathway in HK-2 cells

**Figure 2:**
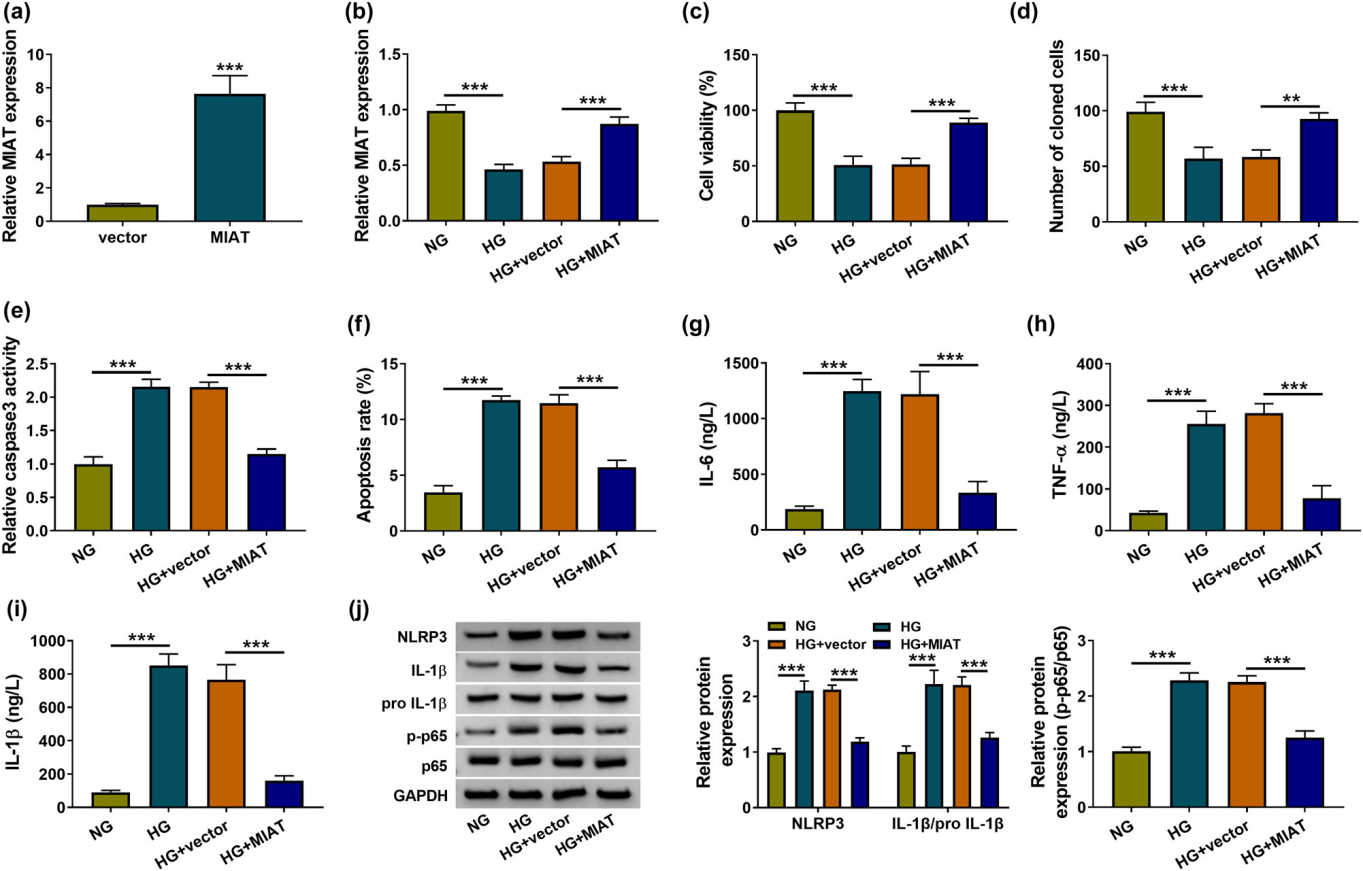
MIAT reestablishment antagonized the HG-induced cell damages and HG-activated NF-κB pathway in HK-2 cells. (a) MIAT expression was detected by RT-qPCR in HK-2 cells transfected with vector or MIAT. (b) The RT-qPCR was applied for MIAT level analysis after treatment of NG, HG, HG + vector, or HG + MIAT in HK-2 cells. (c–f) Cell viability (c), colonizing capacity (d), and apoptosis (e–f) were examined using CCK-8, colony formation assay and caspase-3 activity detection/flow cytometry, respectively. (g–i) The determination of IL-6, TNF-α, and IL-1β was completed by ELISA. (j) Western blot was used to analyze NLRP3, IL-1β/pro-IL-1β, and p-p65/p65 proteins. **P < 0.01, ***P < 0.001.

**Figure A1:**
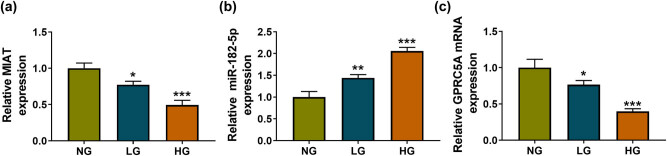
The dysregulation of MIAT, miR-182-5p and GPRC5A after treatment of LG and HG. (a–c) RT-qPCR was performed to examine the levels of MIAT (a), miR-182-5p (b) and GPRC5A mRNA (c) in HK-2 cells with treatment of NG, LG or HG. *P < 0.05, **P < 0.01, ***P < 0.001.

MIAT level was determined by RT-qPCR in glucose-treated HK-2 cells. The data revealed that the expression of MIAT was decreased in LG and HG groups relative to NG group, and the inhibitory effect of HG on MIAT was more significant in HG group than that in LG group (Figure A1a). Furthermore, MIAT vector was constructed for the functional analysis of MIAT in HG-treated HK-2 cells. The RT-qPCR showed that MIAT transfection promoted MIAT expression with 7-fold changes contraposed to vector transfection group in HK-2 cells (Figure 2a) and it also eliminated the HG-induced MIAT downregulation in HK-2 cells (Figure 2b). After MIAT level was upregulated, the HG-mediated inhibitory effects on cell viability (Figure 2c) and colonizing capacity (Figure 2d) together with the stimulative influences on cell apoptosis (Figure 2e–f) and inflammation cytokines levels (Figure 2g–i) were all alleviated at least in part. Also, the inflammation-related NLRP3, IL-1β/pro-IL-1β, and NF-κB-related p-p65/p65 levels were downregulated in HG + MIAT group compared with HG treatment group (Figure 2j). These findings revealed that the reestablishment of MIAT level could inhibit the cell damages and NF-κB signal in HG-treated HK-2 cells.

### 3.3 MIAT directly interacted with miR-182-5p

**Figure 3:**
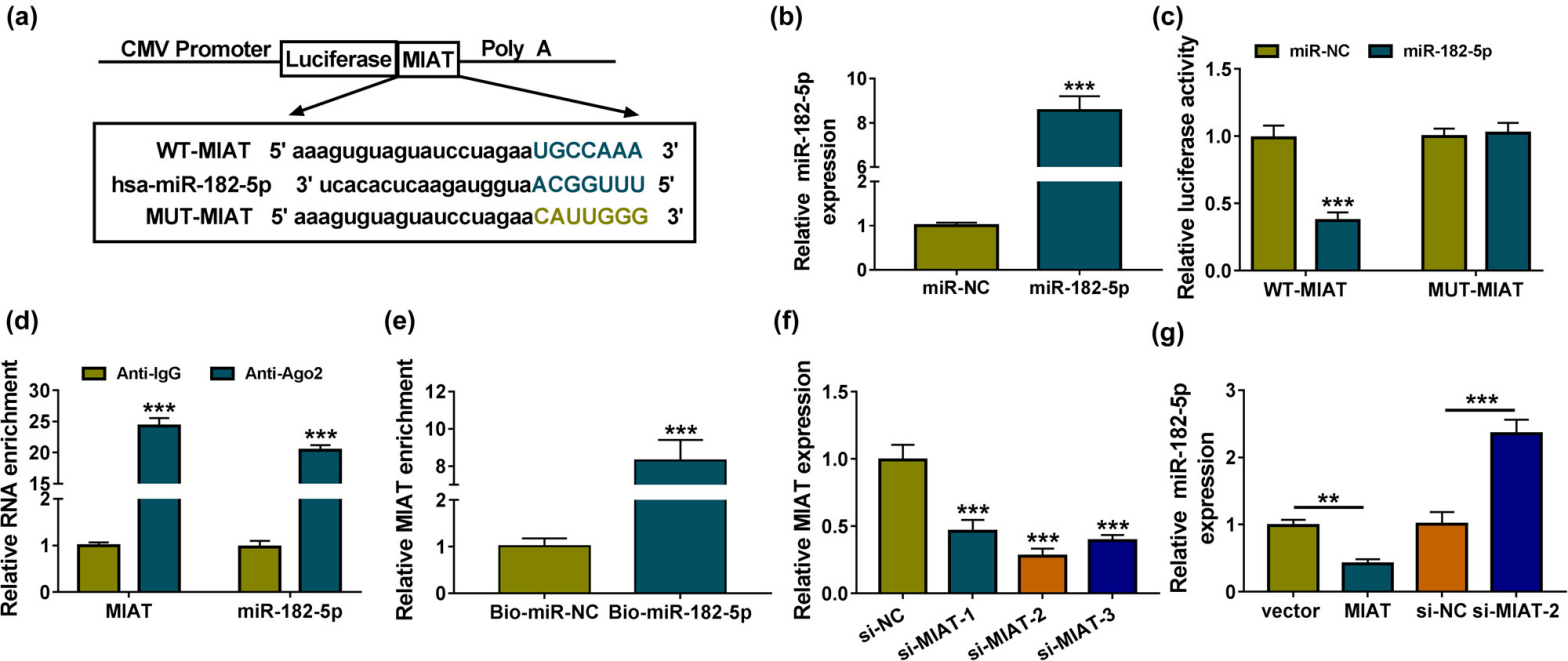
MIAT directly interacted with miR-182-5p. (a) The binding sites between MIAT and miR-182-5p were analyzed via online miRcode. (b) The overexpression efficiency of miR-182-5p mimic was assayed through RT-qPCR. (c–e) Dual-luciferase reporter assay (c), RIP (d), and RNA pull-down assay (e) were conducted to manifest the confirmation between MIAT and miR-182-5p. (f) MIAT detection was carried out using RT-qPCR after transfection of si-NC, si-MIAT-1, si-MIAT-2, or si-MIAT-3. (g) The effects of MIAT and si-MIAT-2 on miR-182-5p were measured by RT-qPCR, with vector and si-NC as the respective control. **P < 0.01, ***P < 0.001.

The online miRcode prediction software exhibited the site binding between the sequences of MIAT (UGCCAAA) and miR-182-5p (ACGGUUU) (Figure 3a), showing the potential of miR-182-5p as a target for MIAT. Compared to MUT-MIAT luciferase reporter plasmid containing the point mutation (CAUUGGG) in miR-182-5p-binding sites, we found that the overexpression of miR-182-5p (Figure 3b) repressed the luciferase intensity of WT-MIAT plasmid in the dual-luciferase reporter assay (Figure 3c). Also, MIAT and miR-182-5p could simultaneously bind to Ago2 protein in RIP assay (Figure 3d). In addition, MIAT was largely captured by Bio-miR-182-5p in comparison to Bio-miR-NC group (Figure 3e). The RT-qPCR manifested that siRNA-mediated suppressive effect on the expression of MIAT was successful and si-MIAT-2 was the most efficient (Figure 3f). MIAT overexpression distinctly reduced the miR-182-5p level, while silence of MIAT induced the opposite influence on miR-182-5p (Figure 3g). All these assays demonstrated the interaction between MIAT and miR-182-5p, and the negative regulation of MIAT on miR-182-5p.

### 3.4 Knockdown of MIAT promoted the HG-induced cell effects and NF-κB signal by upregulating the miR-182-5p level

**Figure 4:**
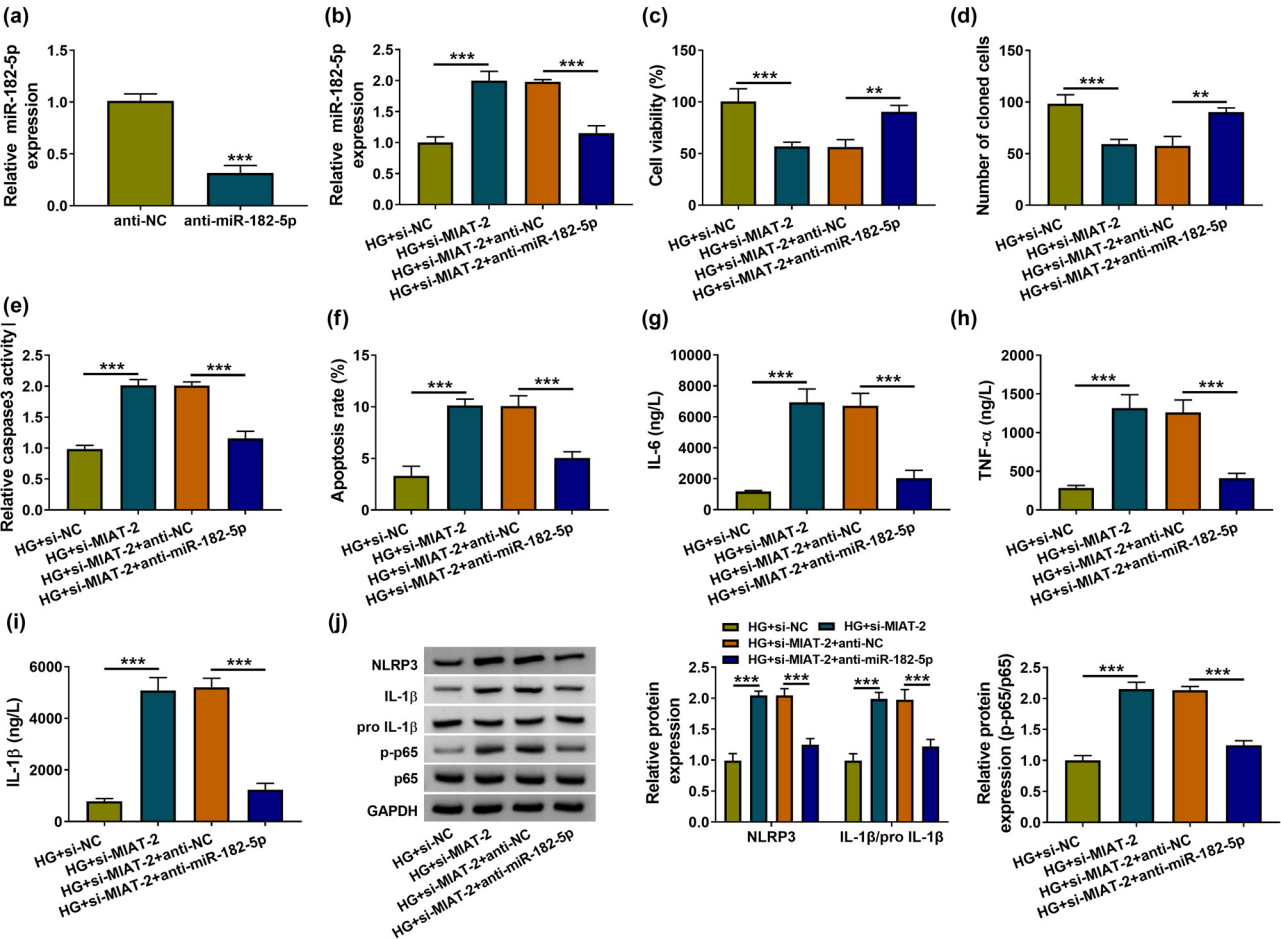
Knockdown of MIAT promoted the HG-induced cell effects and NF-κB signal by upregulating the miR-182-5p level. (a) The influence of anti-miR-182-5p on miR-182-5p was assessed by RT-qPCR. (b) After co-treatment of HG + si-NC, HG + si-MIAT-2, HG + si-MIAT-2 + anti-NC, or HG + si-MIAT-2 + anti-miR-182-5p, RT-qPCR was performed for examining the expression of miR-182-5p. (c–f) The analyses of cell viability (c), colony formation (d), and apoptosis (e–f) were completed by implementing CCK-8, colony formation assay, and caspase-3 assay/flow cytometry. (g–i) Inflammatory cytokines were measured via ELISA. (j) The inflammation and NF-κB associated proteins were determined via western blot. **P < 0.01, ***P < 0.001.

**Figure A2:**
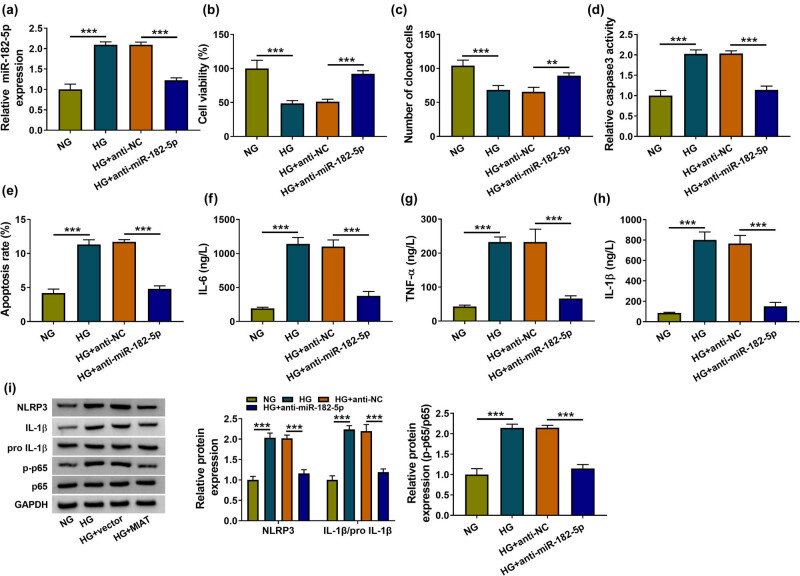
Inhibition of miR-182-5p reduced the HG-induced toxicity in HK-2 cells. HK-2 cells were treated with NG, HG, HG + anti-NC or HG + anti-miR-182-5p. (a) The miR-182-5p expression was determined by RT-qPCR. (b–c) Cell viability (b) and colonizing capacity (c) were respectively measured using CCK-8 and colony formation assay. (d–e) Caspase 3 assay (d) and flow cytometry (e) were performed to assess cell apoptosis. (f–h) The concentrations of inflammatory cytokines were detected via ELISA. (i) The proteins of inflammation and NF-*κ*B pathway were assayed by western blot. **P < 0.01, ***P < 0.001.

RT-qPCR demonstrated that miR-182-5p level was increasingly elevated in LG and HG groups in contrast with NG group (Figure A1b). The transfection of anti-miR-182-5p has reduced the miR-182-5p expression in HK-2 cells compared to anti-NC transfection (Figure 4a). The functional analysis has demonstrated that the inhibition of miR-182-5p could reduce all the HG-triggered toxicity in HK-2 cells (Figure A2). To explore whether the function of MALAT1 in regulating cell injury was related to miR-182-5p, HG-treated HK-2 cells were transfected with si-NC, si-MIAT-2, si-MIAT-2 + anti-NC, and si-MIAT-2 + anti-miR-182-5p. The RT-qPCR showed that the upregulation of miR-182-5p level caused by si-MIAT-2 was attenuated following the inhibition of miR-182-5p in HG-treated cells (Figure 4b). MIAT downregulation reduced cell viability (Figure 4c) and colony formation ability (Figure 4d), but promoted cell apoptosis (Figure 4e–f) and inflammatory cytokine release (Figure 4g–i) in HG-treated HK-2 cells, whereas all these effects were abolished by miR-182-5p inhibitor. The protein levels of NLRP3, IL-1β/pro-IL-1β, and p-p65/p65 were all markedly higher in HG + si-MIAT-2 group than those in HG + si-NC group, while the downregulation of miR-182-5p returned the si-MIAT-2-mediated exacerbation of inflammation and activation of NF-κB pathway (Figure 4j). From the above results, we could find that cell damages and NF-κB signal activation induced by HG were enhanced by MIAT downregulation to elevate the level of miR-182-5p.

### 3.5 MIAT acted as miR-182-5p sponge to promote GPRC5A expression

**Figure 5:**
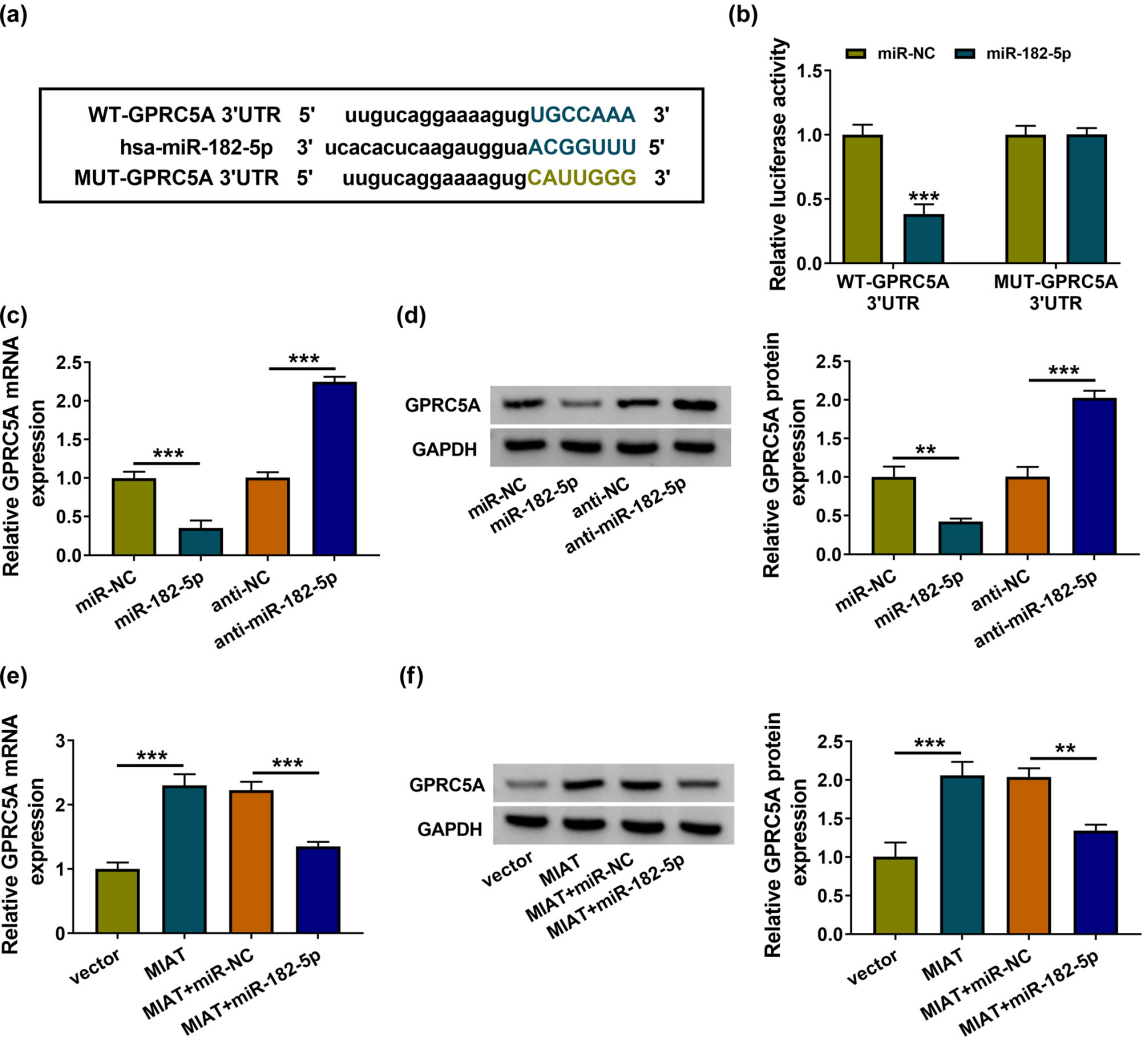
MIAT acted as an miR-182-5p sponge to promote GPRC5A expression. (a) TargetScan was used for the bioinformatics analysis between miR-182-5p and GPRC5A 3′-UTR. (b) The binding of miR-182-5p with GPRC5A 3′-UTR was validated via the dual-luciferase reporter assay. (c and d) The RT-qPCR and western blot were administrated to assay the mRNA (c) and protein (d) expression of GPRC5A after transfection of miR-182-5p, anti-miR-182-5p, or the matched control groups. (e and f) GPRC5A detection was executed by RT-qPCR and western blot in vector, MIAT, MIAT + miR-NC, or MIAT + miR-182-5p transfection group. **P < 0.01, ***P < 0.001.

TargetScan predicted that there were binding sites for miR-182-5p in GPRC5A 3′-UTR sequence (Figure 5a). Dual-luciferase reporter assay indicated that luciferase activity of WT-GPRC5A 3′-UTR was reduced by miR-182-5p, but the luciferase activity of MUT-GPRC5A 3′-UTR was not affected (Figure 5b). GPRC5A mRNA and protein levels were respectively downregulated or upregulated after the overexpression or inhibition of miR-182-5p in normal HK-2 cells (Figure 5c and d), revealing that miR-182-5p negatively regulated the expression of GPRC5A. Furthermore, MIAT overexpression has upregulated the mRNA and protein levels of GPRC5A, while miR-182-5p mimic eliminated the regulation of MIAT on GPRC5A (Figure 5e and f). Thus, MIAT could promote the expression of GPRC5A via sponging miR-182-5p.

### 3.6 GPRC5A upregulation suppressed the miR-182-5p-mediated cell damages and NF-κB signal in HG-treated HK-2 cells

**Figure 6:**
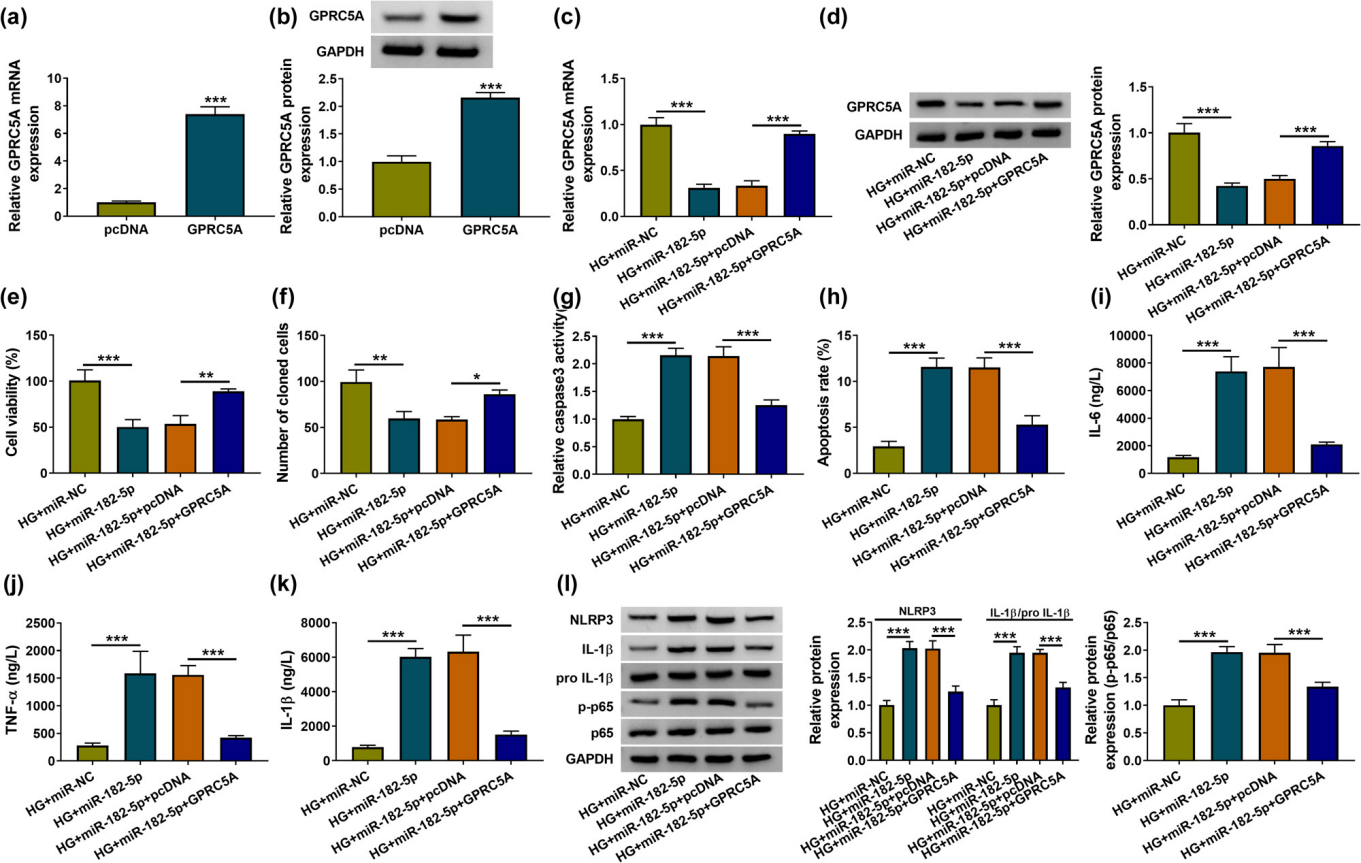
GPRC5A upregulation suppressed the miR-182-5p-mediated cell damages and NF-κB signal in HG-treated HK-2 cells. (a and b) The efficiency of GPRC5A vector was analyzed via RT-qPCR and western blot. (c and d) After HK-2 cells were treated with HG + miR-182-5p, HG + miR-182-5p + GPRC5A, or the relative controls, the RT-qPCR and western blot were conducted to examine the GPRC5A expression. (e–k) CCK-8 for cell viability (e), colony formation assay for clonal ability (f), caspase-3 assay/flow cytometry for cell apoptosis (g and h) and ELISA for inflammation (i–k) were used for the assessment of the different transfections on biological processes. (l) Western blot was administrated to determine the protein levels related to inflammation and NF-κB pathway. *P < 0.05, **P < 0.01, ***P < 0.001.

The expression analysis for GPRC5A has manifested that the mRNA level of GPRC5A was downregulated after treatment of LG or HG in comparison to the treatment of NG in HK-2 cells, and the HG-induced expression change was more obvious than LG (Figure A1c). GPRC5A overexpression vector was constructed to analyze the effects of miR-182-5p and GPRC5A on DN. The transfection efficiency of GPRC5A was great in HK-2 cells (Figure 6a and b). HK-2 cells were then treated with HG + miR-NC, HG + miR-182-5p, HG + miR-182-5p + pcDNA, or HG + miR-182-5p + GPRC5A. The introduction of GPRC5A lightened the expression inhibition of GPRC5A mRNA and protein caused by miR-182-5p, also exhibiting the successful overexpression of GPRC5A vector (Figure 6c and d). CCK-8 and colony formation assays manifested that cell viability (Figure 6e) and cloned cells (Figure 6f) were inhibited by miR-182-5p mimic in HK-2 cells treated with HG, while this inhibition was abated after the upregulation of GPRC5A. The same reversal of GPRC5A overexpression was found in miR-182-5p-induced promotion of caspase-3 activity (Figure 6g) and apoptosis rate (Figure 6h) in HG-treated HK-2 cells. The introduction of miR-182-5p also facilitated the IL-6, TNF-α, and IL-1β levels, but the following GPRC5A transfection weakened the inflammation promotion triggered by miR-182-5p (Figure 6i and k). Also, miR-182-5p enhanced the protein expression of NLRP3, IL-1β/pro-IL-1β, and p-p65/p65 to activate cell inflammation and NF-κB pathway via inhibiting the level of GPRC5A in HG-induced DN cell model (Figure 6l). Collectively, miR-182-5p also aggravated the HG-triggered HK-2 cell damages and accelerated the HG-induced NF-κB pathway by downregulating GPRC5A.

## 4 Discussion

DN is a progressive disorder with great threat for diabetic patients, and lncRNAs have recently become the research emphasis in DN [26]. In this study, we provided interesting evidence for the involvement and functional mechanism of MIAT in DN progression.

Dysfunction of renal tubule is the initial event for DN and renal tubular epithelial cells have crucial functions in DN [27]. In the renal tubular epithelial cells (HK-2), we found that HG treatment generated a suppressive effect on cell growth and a promoting effect on cell apoptosis. By detecting the representative inflammatory cytokines (IL-6, TNF-α, and IL-1β), HG was observed to induce the inflammatory response in HK-2 cells. NLRP3 inflammasome plays a significant role in the initiation of inflammation to evoke the release of inflammatory cytokines [28]. Cellular NF-κB pathway can be activated in the process of inflammatory response of DN [29]. Our western blot results indicated that NLRP3, IL-1β, and NF-κB p-p65 protein levels were all enhanced in HG-treated HK-2 cells, insinuating that HG induced cell inflammation and activated the NF-κB pathway.

Mounting studies have suggested the correlations of lncRNAs with diabetic complications. For instance, MALAT1 enhanced the HG-induced cartilage endplate cell apoptosis by the p38/MAPK signaling pathway [30] and CASC15 promoted diabetes-induced chronic renal failure and podocyte apoptosis [31]. It has been found that MIAT facilitated diabetic retinopathy via activating TGF-β1 signaling [32] and induced cardiomyocyte apoptosis in diabetic cardiomyopathy by targeting the miR-22-3p/DAPK2 axis [33]. lncRNA-Gm4419 knockdown inhibited the NF-κB pathway and inflammatory response in DN [34]. Our analysis exhibited that MIAT impeded the HG-induced cell damages and HG-activated NF-κB pathway *in vitro*. First, we found that the reestablishment of MIAT expression elevated cell viability in HG-stimulated HK-2 cells and this result was in accordance with the previous study [15]. In conformity to the issued findings of MIAT [16], we furthered affirmed the proliferative effect of MIAT on HG-treated HK-2 cells using colony formation assay. In addition, cell apoptosis and inflammation were repressed after the upregulation of MIAT. Zhang et al. stated that MIAT contributed to the HG-induced apoptosis and inflammation in podocytes to accelerate the DN progression [35]. On the contrary, the current data suggested that MIAT functioned as an inhibitor in the development of DN.

lncRNAs can affect gene expression and the downstream signaling pathways by functioning as the sponges of miRNAs [36,37]. Here miR-182-5p was first affirmed as a miRNA target of MIAT and the functional regulation of MIAT on DN or NF-κB pathway was achieved by targeting miR-182-5p in HG-treated HK-2 cells. For miRNAs, miR-451 repressed the NF-κB-mediated inflammatory response in DN by downregulating LMP7 [38] and miR-140-5p lightened HG-induced apoptosis or inflammation via inhibiting the TLR4/NF-κB signaling pathway in HK-2 cells [39]. We have ensured that miR-182-5p targeted GPRC5A to regulate the expression of GPRC5A, negatively. Furthermore, miR-182-5p enhanced the DN progression and NF-κB signal in HG-treated HK-2 cells by targeting GPRC5A. The inhibitory effect of GPRC5A on NF-κB pathway has been discovered in lung inflammation [40]. PVT1 facilitated the HG-induced mesangial cell proliferation and fibrosis via sponging miR-23b-3p to result in the regulation of WT1 [41]. MALAT1 enhanced the DN progression in renal tubular epithelial cells by increasing NLRP3 level through playing the sponge role for miR-30c [42]. Ji et al. have revealed that MIAT served as a miR-147a sponge to regulate E2F3 in DN [16]. MIAT was also involved in the pathogenesis of DN by sponging miR-130a-3p and regulating TLR4 [35]. Herein our data revealed that MIAT could affect the expression of GPRC5A via the sponge effect on miR-182-5p. Thus, this study provided direct evidence for the presence of MIAT/miR-182-5p/GPRC5A axis in the regulation of DN progression and NF-κB pathway.

However, this study has some limitations. For example, the dose scope of glucose treatment was not wide. Zhou et al. have found that cell viability reduction and MIAT expression downregulation were more significant after 30 or 45 mM of glucose treatment [15]. Exploring whether MIAT or GPRC5A overexpression and miR-182-5p downregulation can antagonize the effects of higher dose of glucose on HK-2 cells is also important. In addition, all experiments were conducted in cell line *in vitro*. It is necessary to investigate the expression level of MIAT in clinical samples (or primary cells) and the correlation among MIAT, miR-182-3p, and GPRC5A. Moreover, the research of MIAT/miR-182-5p/GPRC5A axis *in vivo* remains to be performed in future.

## 5 Conclusion

**Figure 7:**
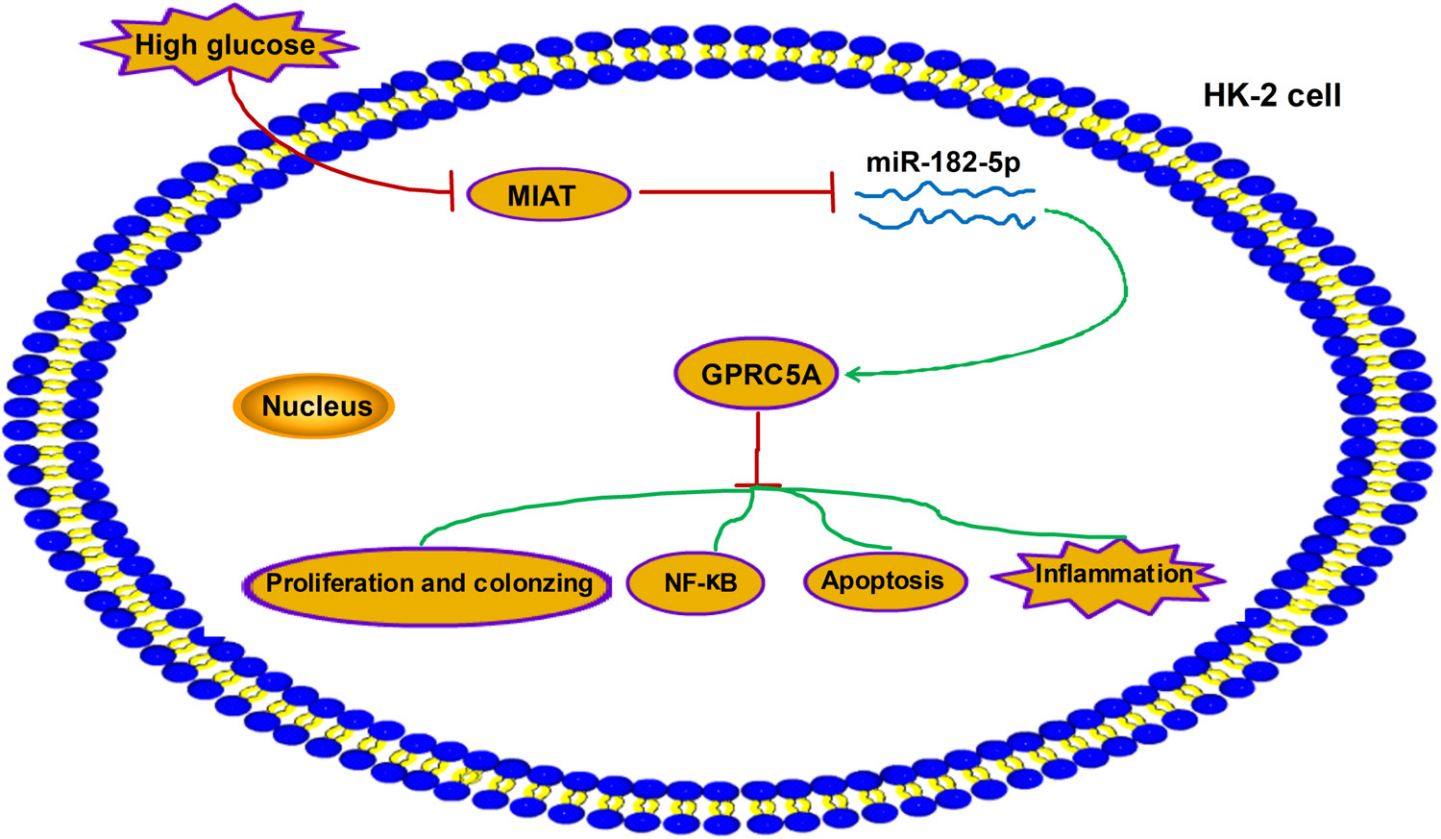
MIAT regulated the HG-induced effects on cell proliferation, colonizing, apoptosis, and inflammation via the miR-182-5p/GPRC5A-mediated NF-κB pathway.

To conclude, lncRNA MIAT could serve as a sponge of miR-182-5p to promote GPRC5A expression to further affect the NF-κB pathway and biological behaviors (proliferation, colonizing, apoptosis, and inflammation) in HG-treated HK-2 cells (Figure 7). MIAT inhibited the NF-κB pathway and DN progression *in vitro* via the mediation of miR-182-5p/GPRC5A network, and MIAT might have considerable therapeutic value for DN.
